# Pathophysiological mechanisms for the respiratory syncytial virus-reactive airway disease link

**DOI:** 10.1186/rr185

**Published:** 2002-06-24

**Authors:** Giovanni Piedimonte

**Affiliations:** 1 Departments of Pediatrics, Medicine, and Molecular/Cellular Pharmacology, University of Miami School of Medicine, Miami, Florida, USA

**Keywords:** asthma, palivizumab, pediatrics, prevention, reactive airway disease, respiratory syncytial virus

## Abstract

There is substantial epidemiological evidence supporting the concept that respiratory syncytial virus (RSV) lower respiratory tract infection in infancy may be linked to the development of reactive airway disease (RAD) in childhood. However, much less is known concerning the mechanisms by which this self-limiting infection leads to airway dysfunction that persists long after the virus is cleared from the lungs. A better understanding of the RSV–RAD link may have important clinical implications, particularly because prevention of RSV lower respiratory tract infection may reduce the occurrence of RAD later in life. Among the mechanisms proposed to explain the chronic sequelae of RSV infection is the interaction between the subepithelial neural network of the airway mucosa and the cellular effectors of inflammatory and immune responses to the virus. The body of clinical literature linking RSV and RAD is reviewed herein, as are the cellular and molecular mechanisms of neuroimmune interactions and neural remodeling that may underlie this link, and the possibility that preventing the infection may result in a decreased incidence of its chronic sequelae.

## Introduction

Respiratory syncytial virus (RSV) is the most important respiratory pathogen in infancy and can cause serious lower respiratory tract infections (LRTIs), particularly in prematurely born infants and children with underlying cardiorespiratory conditions [1]. Epidemiologic studies [2-9] have indicated that RSV LRTI in infancy is associated with recurrent wheezing later in life, giving rise to the theory that there is a link between RSV and reactive airway disease (RAD). The pathogenetic mechanisms of RSV-induced airway inflammation and hyperreactivity remain largely unknown, however, and no effective therapeutic option is currently available to manage the acute and chronic clinical manifestations of this infection. The present review focuses on the epidemiologic evidence linking RSV and RAD, and on the cellular and molecular mechanisms of airway inflammation during and after RSV infection.

## RSV LRTI: epidemiology and long-term consequences

The majority of children will become infected with RSV by the time they are 2 years old [10]. A link between RSV LRTI in infancy and later development of RAD in childhood has been suggested on the basis of several retrospective studies (Table 1) [2-9]. In addition, two recent prospective studies [11,12], which are described below, have confirmed that RSV is a major risk factor for the development of recurrent wheezing and RAD during the first decade of life.

Sigurs *et al*. [11] conducted a prospective study of 47 previously healthy infants hospitalized for RSV bronchiolitis during their first year of life and a reference population of 93 infants with no history of RSV infection. The infants in the two groups were well matched for age, sex, family history of RAD or atopy, and general living environment. RAD, wheezing, and recurrent wheezing were the outcome measures. A diagnosis of RAD required having had three or more episodes of physician-diagnosed bronchial obstruction; recurrent wheezing was defined as three or more episodes of self-reported bronchial obstruction; and wheezing was defined as any episode of bronchial obstruction. The patient's allergic status was determined with skin prick testing and titers of serum IgE antibodies.

By 7 years of age 30% of children in the RSV group had experienced RAD at some time, versus only 3% of children in the reference group (Table 2) [11]. Likewise, 23% of children in the RSV group had physician-diagnosed RAD at age 7 years as compared with 2% of children in the reference group. The cumulative prevalence of wheezing among children in the RSV group was twice that observed in the reference group (68% versus 34%, respectively) and the presence of any wheezing at age 7 years was 38% in the RSV group and 2% in the control group. RSV bronchiolitis was the only significant risk factor for RAD, whereas RSV bronchiolitis, family history of atopy, and male sex were all risk factors for 'any wheezing'. Multivariate analysis showed that the highest frequency of RAD occurred when both RSV bronchiolitis and a family history of atopy were present as risk factors. That study also found a link between RSV bronchiolitis and atopy; this has not been observed in other studies, possibly due to differences in the severity of RSV disease and/or genetic background among the populations examined.

**Figure 1:**
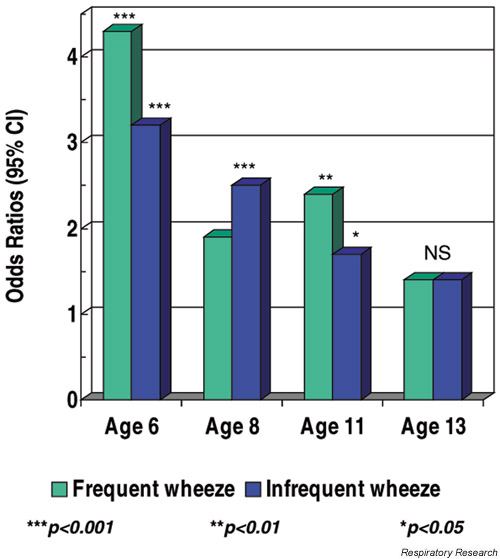
Risk of frequent and infrequent wheeze in children who had mild to moderate respiratory syncytial virus lower respiratory tract infection [12]. CI, confidence interval; NS, not significant.

Stein *et al*. [12] studied infants enrolled in the Tucson Children's Respiratory Study who developed mild to severe RSV disease without necessarily requiring hospitalization. The study design required parents to complete questionnaires when the children were 6, 8, 11, and 13 years old. A total of 472 out of 519 children who had at least one LRTI during the first 3 years of life were tested for viruses, and 207 of these 472 children had documented RSV infections. It was found that children with a history of RSV LRTI were 3.2 times more likely to have infrequent wheeze and 4.3 times more likely to have frequent wheeze by age 6 years (Fig. 1). However, by the time these children were 13 years old the association between wheeze and RSV LRTI was no longer statistically significant.

Another interesting finding was that, although children who experienced RSV infection had significantly lower forced expiratory volume in 1 s, they were also significantly more responsive to bronchodilation with albuterol than were the reference group [12]. This points to decreased pulmonary function being caused by reversible dysregulation of airway tone. Skin tests and serum IgE concentrations showed no link between RSV infection and atopy. Overall, the results of that study confirmed that when RSV infection occurs in children by age 3 years it is linked to a significantly increased risk for recurrent wheezing during the first decade of life.

## Cellular and molecular mechanisms linking RSV to RAD

Various theories have been put forth in an attempt to explain how RSV generates long-term airway inflammation and hyperreactivity. We have proposed that interactions between neural and immunoinflammatory mechanisms may cause inflammation well after the initial RSV infection is cleared. The discussion below addresses the interplay between these mechanisms and how this may lead to recurrent cycles of airway inflammation.

### Neurogenic inflammation

An animal model of RSV bronchiolitis was developed in rat strain Fischer F-344 [13]; these rats mount a strong immune response and are thus able to clear RSV from the lungs within few weeks. This is quite similar to the self-limiting infection caused by RSV in immunocompetent children. In addition, the inoculation of weanling rats with RSV has allowed us to examine the long-term effects of early RSV infection [14].

Our first studies in this model focused on the nonadrenergic noncholinergic (NANC) nervous system and its role in the modulation of local inflammatory and immune responses in the airways. In particular, the excitatory component of this NANC system (NANCe) is able to sense the physical and chemical changes that arise in respiratory epithelium exposed to airborne irritants, and sends to the central nervous system the nociceptive information that starts the cough reflex. In addition, NANCe nerves release substance P and other peptide neurotransmitters at the site of stimulation, playing an important role in the initial phase of the inflammatory process and in the modulation of the immune response to the virus.

We have found that RSV upregulates the expression of the gene that encodes the NK1 receptor subtype, which mediates the inflammatory and immunomodulatory effects of substance P [13,14], thereby potentiating neurogenically mediated airway inflammation. In contrast, RSV does not affect the NK2 receptor subtype, which mediates airways smooth muscle contraction, suggesting that airflow obstruction during RSV infection is more likely to be due to mucosal inflammatory edema than to bronchoconstriction.

In the animal model, developmental changes in the distribution of neurogenic inflammatory responses across the respiratory tract were observed as the rats evolved from infancy to adulthood; strong neurogenically mediated inflammation developed in the lower respiratory tract of young rats, whereas in adult rats this response was confined to the large extrapulmonary airways. This observation can be explained by a higher density and/or responsiveness of NANCe nerve fibers distributing to the lower airways during infancy, and provides a potential mechanism to explain why RSV bronchiolitis tends to occur exclusively in infants, whereas infection with this virus usually manifests as an upper respiratory tract infection in older persons.

### Lymphocytes

Our recent research has suggested that RSV infection may also induce the expression of NK1 receptors in T-lymphocyte subpopulations within the bronchiolar-associated lymphoid tissue. These NK1-receptor-bearing lymphocytes are in essence 'primed' by the virus to be deployed into the airways following nerve stimulation by airborne irritants and can also be activated for the release of proinflammatory cytokines. CD4^+^ T-helper cells appear to be preferentially affected by the chemotactic activity of substance P, which leads to accumulation of these cells in the infected airways.

### Mast cells

In a recent study [15] we showed that acute RSV infection is associated with marked increase in the number of mast cells in the airway mucosa. RSV also increased expression of the 5-lipoxygenase gene, with transient production of cysteinyl leukotrienes, probably deriving from the expanded mast cell population found in infected lungs. Interestingly, the mast cells in the infected mucosa appeared to form clusters around nerve fibers, suggesting functional interactions through the formation of local neuron–mast cell feedback loops. In fact, the leukotriene receptor antagonist montelukast potently inhibited neurogenically mediated inflammation in the intrapulmonary airways of infected weanling rats, and to a much lesser degree in adult rats. No significant inhibitory effect was found in the extrapulmonary airways. These findings suggest that leukotrienes are released via mast cell–nerve interactions, which are amplified during RSV infections and in turn potentiate the inflammatory effects of neuropeptides such as substance P. If the same mechanisms are at work in human airways then leukotriene modifiers could be beneficial in the therapy of RSV bronchiolitis, and controller therapy for pediatric asthma with leukotriene modifiers may protect against virus-induced asthma exacerbations.

## RSV-induced neural remodeling

Another recent study from our laboratory [16] showed that aging is associated with a progressive decline in the expression of the prototypical neurotrophin nerve growth factor (NGF) in the lungs, which is paralleled by a similar decline in the expression of its high-affinity (trkA) and low-affinity (p75) receptors. RSV infection interferes with this physiologic decline, promoting a large increase in the expression of both NGF and neurotrophin receptors. The lungs of weanling rats not only have markedly higher baseline expression of neurotrophic factors but also appear to be more responsive to the perturbation introduced by the infecting virus. Furthermore, our data indicate that NGF upregulates expression of the NK1 receptor, which mediates the inflammatory effects of substance P and is responsible for the exaggerated neurogenic inflammation in RSV-infected airways. Accordingly, selective NGF blockade inhibits neurogenically mediated inflammation during RSV infection, and the magnitude of this inhibitory effect is more prominent in younger animals. Thus, RSV-induced release of NGF may lead to short-term and long-term changes in the distribution and reactivity of sensory nerves across the respiratory tract, contributing toward exaggerated inflammatory reactions during and after the infection. NGF and its receptors may also amplify other immunoinflammatory and neuronal pathways that contribute to airway inflammation and hyperreactivity. This process of virus-dependent neural remodeling appears to be particularly extensive when the infection occurs early in life, because of the much higher degree of neural plasticity that characterizes infancy.

**Figure 2:**
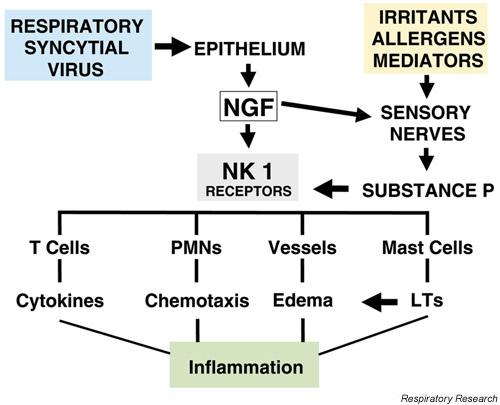
Neuroinflammatory interactions and neural remodeling in airways infected with respiratory syncytial virus. LTs, leukotrienes; NGF, nerve growth factor; PMNs, polymorphonuclear leukocytes.

In summary, we propose that remodeling of the submucosal neural network and the deriving cluster of neuroimmune interactions may link RSV infections that occur during critical developmental 'windows' with RAD in childhood. Based on this model, activation of the upregulated NANCe system by irritants would be responsible for the recurring airway inflammation and subsequent narrowing, which continues after the acute RSV infection has cleared (Fig. 2). If this is the case then it is possible that inhibition of RSV penetration or replication in the lower respiratory tract could reduce the frequency of childhood RAD.

## Treating RSV

There are currently no effective treatments for acute RSV infection, and there is little clinical evidence to indicate that antiviral therapy will reduce the risk for developing RAD. Results obtained with ribavirin were reviewed in order to determine whether treatment helped to avoid long-term sequelae [17]. In three controlled studies with long-term follow up, no differences were observed between ribavirin-treated and placebo-treated children with respect to the incidence of long-term sequelae, RAD, recurrent LRTI, and wheezing. The one study that reported a reduction in the incidence of post-RSV RAD followed children for only 1 year, thus giving no real indication of whether antiviral therapy has long-term beneficial effects in this regard.

Passive immunoprophylaxis is currently the only option for avoiding RSV disease. IMpact-RSV [18] was a randomized, double-blind, placebo-controlled trial conducted at 139 sites in the USA, the UK, and Canada. It evaluated prophylaxis with palivizumab, a humanized monoclonal antibody that is specific for the RSV fusion protein, in 1502 prematurely born children with or without chronic lung disease (CLD). Children received an intramuscular injection of either palivizumab (15 mg/kg) or placebo every month for 5 months. An overall 55% reduction in hospitalizations for RSV was observed as the primary endpoint of this study (10.6% in the placebo group versus 4.8% in the palivizumab group). Specifically, prematurely born children without CLD had a 78% reduction in RSV-related hospitalizations (8.1% versus 1.8%) as compared with a 39% reduction (12.8% versus 7.9%) for children with CLD.

These results, coupled with the epidemiologic evidence of the link between RSV and RAD, raise the issue of whether immunoprophylaxis against RSV can reduce the risk for development of RAD. In our rat model, prophylaxis with palivizumab prevented the development of acute neurogenic inflammatory changes in the lower respiratory tract following inoculation of RSV [19]. Palivizumab prophylaxis also appeared to guard against development of long-term vulnerability to neurogenically mediated inflammation.

This issue was also approached in a study of children with CLD (*n* = 13) who had received RSV immune globulin 7–10 years earlier [20]. Pulmonary function was significantly better in the treatment group than in matched control patients (*P* < 0.02). Significantly less atopy (*P* = 0.04) and a lower likelihood of RAD attacks (*P* = 0.03) were observed. Based on this preliminary evidence, the investigators suggested that RSV prophylaxis might have long-term benefit in reducing the risk for RAD.

Being 50–100 times more potent than RSV immune globulin, palivizumab may be an important alternative in the prophylaxis of RSV and the avoidance of RAD. A prospective, multicenter, case–control study to examine the effect of palivizumab prophylaxis on the incidence and degree of reversible airway obstruction in premature children with RSV is ongoing in Europe (Protocol W00-353; Abbott Laboratories, Abbott Park, IL, USA). It is anticipated that 300 children will be enrolled and divided evenly between three study arms; 100 children who receive palivizumab prophylaxis will be compared with 100 children who had documented RSV bronchiolitis and with 100 control children. Follow up will continue for 3 years using monthly telephone calls and scheduled visits at 6, 12, 24, and 36 months. The end-points include results from a respiratory questionnaire, history of wheezing, inventory of medications used, record of hospitalizations, and pharmacoeconomic evaluation. Results from the study are anticipated at the end of 2003.

## Conclusion

There is mounting evidence suggesting that young children who contract RSV infection are more likely to suffer from long-term respiratory sequelae, such as RAD, later in life. This link has been supported by well controlled epidemiologic studies, and data from animal models have identified molecular mechanisms that can explain the effects of RSV infection on airway inflammation and hyperreactivity. Preventing RSV is therefore a promising approach to reducing the incidence of RAD in childhood. Studies evaluating the long-term benefits of RSV prophylaxis using agents such as palivizumab will help to determine the validity of this hypothesis.

## Abbreviations

CLD = chronic lung disease; LRTI = lower respiratory tract infection; NANC(e) = nonadrenergic noncholinergic (excitatory component); NGF = nerve growth factor; RAD = reactive airway disease; RSV = respiratory syncytial virus.

## Figures and Tables

**Table 1:** Retrospective studies showing an association between respiratory syncytial virus infection and reactive airway disease

Reference	Year	Patients with RSV/total (*n*)^*^	Years of follow up	*P* (odds ratio)
[2]	1978	35/90	8	<0.02
[3]	1982	130/241	10	<0.001
[4]	1984	59/236	8	<0.0001
[5]	1984	100/300	7	<0.001
[6]	1987	51/75	2	<0.01
[7]	1992	73/146	5.5	<0.001
[8]	1993	70/140	1	0.001
[9]	1997	61/108	9–10	(3.59)

^*^The remainder of the patients were in the control group. RSV, respiratory syncytial virus.

**Table 2:** Cumulative percentages of children with asthma, recurrent wheezing, or any wheezing at age 7.5 years

Symptom	RSV group (*n* = 47)	Control group (*n* = 93)	*P*
Asthma	30%	3%	<0.0001
Recurrent wheezing	28%	11%	0.015
Any wheezing	68%	34%	<0.001

Data from Sigurs *et al*. [11]. RSV, respiratory syncytial virus.
